# Heat-Affected Zone and Mechanical Analysis of GFRP Composites with Different Thicknesses in Drilling Processes

**DOI:** 10.3390/polym13142246

**Published:** 2021-07-08

**Authors:** Usama A. Khashaba, Mohamed S. Abd-Elwahed, Ismai Najjar, Ammar Melaibari, Khaled I. Ahmed, Redouane Zitoune, Mohamed A. Eltaher

**Affiliations:** 1 Mechanical Engineering Department, Faculty of Engineering, King Abdulaziz University, Jeddah 22254-2265, Saudi Arabia; khashabu@zu.edu.eg (U.A.K.); msabdelwahed@gmail.com (M.S.A.-E.); najjar@kau.edu.sa (I.N.); aamelaibari@kau.edu.sa (A.M.); kahmed@kau.edu.sa (K.I.A.); 2 Mechanical Design and Production Engineering Department, Faculty of Engineering, Zagazig University, Zagazig 44519, Egypt; 3 K. A. CARE Energy Research and Innovation Center, King Abdulaziz University, Jeddah 22254-2265, Saudi Arabia; 4 Institut Clément Ader (ICA), UMR-CNRS 5312, “INSA, UPS, Mines Albi, ISAE”, 31400 Toulouse, France; Rzitoune2000@yahoo.fr

**Keywords:** thermal analysis, failure assessments, woven glass fiber composites, drilling of composite, optimization, response surface methodology

## Abstract

This article presents a comprehensive thermomechanical analysis and failure assessment in the drilling of glass fiber-reinforced polymer (GFRP) composites with different thicknesses using a CNC machine and cemented carbide drill with a diameter of 6 mm and point angles of *ϕ* = 118°. The temperature distribution through drilling was measured using two techniques. The first technique was based on contactless measurements using an IR Fluke camera. The second was based on contact measurements using two thermocouples inserted inside the drill bit. A Kistler dynamometer was used to measure the cutting forces. The delamination factors at the hole exit and hole entry were quantified by using the image processing technique. Multi-variable regression analysis and surface plots were performed to illustrate the significant coefficients and contribution of the machining variables (i.e., feed, speed, and laminate thickness) on machinability parameters (i.e., the thrust force, torque, temperatures, and delamination). It is concluded that the cutting time, as a function of machining variables, has significant control over the induced temperature and, thus, the force, torque, and delamination factor in drilling GFRP composites. The maximum temperature recorded by the IR camera is lower than that of the instrumented drill because the IR camera cannot directly measure the tool–work interaction zone during the drilling process. At the same cutting condition, it is observed that by increasing the thickness of the specimen, the temperature increased. Increasing the thickness from 2.6 to 7.7 had a significant effect on the heat distribution of the HAZ. At a smaller thickness, increasing the cutting speed from 400 to 1600 rpm decreased the maximum thrust force by 15%. The push-out delaminations of the GFRP laminate were accompanied by edge chipping, spalling, and uncut fibers, which were higher than those of the peel-up delaminations.

## 1. Introduction

Fiber-reinforced polymer (FRP) composites have desirable features such as design flexibility, low weight, high strength, and a high stiffness-to-weight ratio. These features have allowed FRP to be recommended as structural parts in the aircraft and spacecraft industries, railway, automobile, aeronautical, and marine vehicles, pressure vessels, sporting goods, wind energy, and mechanical and plant engineering [1,2]. In these applications, drilling holes are essential for repairing composite structures and in the assembly/fabrication of composite structures. For example, as one of the leaders of aircraft manufacturing, the Airbus company produced over 120 million holes for assembling 630 A320 family aircraft [3].

Laminated composite structures are made up of composite material plies with desirable angle orientations to accomplish desirable and high-performance mechanical properties [4]. FRP is used to enhance the chemical corrosion resistance and local buckling resistance of concrete-filled steel tube columns [5,6,7]. Drilling FRP laminates with a twist or special drill bits remained the most frequently and economically used machining operation in the industry [8]. Due to the heterogeneity and anisotropy of FRP laminates, they have become one of the typical difficult-to-machine materials [9]. The drilling procedure of a composite is a common machining operation, which is still an open problem for academics and industry. 

In 1990, Ho-Cheng et al. [10] and Tagliaferri et al. [11] investigated and predicted the damage zone and delamination of a laminated composite induced during drilling using a fracture mechanics approach. Khashaba et al. [12] examined the influence of drilling parameters on cutting forces and torques in drilling chopped composites and predicted that the delamination size decreased with the decreasing feed and is insignificantly affected by the cutting speed. Shyha et al. [13] evaluated the effect of the drill geometry and drilling conditions on the tool life and hole quality of an unbacked carbon CFRP laminate. Khashaba et al. [14,15] concluded that the critical thrust force of drilling is affected by the drill pre-wear and surface roughness profile due to burning the matrix. Palanikumar [16] and Rajmohan and Palanikumar [17] optimized the drilling parameters such as the thrust force, workpiece surface roughness, and the delamination factor by considering multiple regression. Palanikumar and Muniaraj [18] experimentally studied the thrust in the drilling of cast hybrid metal matrix (Al–15%SiC–4%graphite) composites using tin-coated solid carbide drills. Nasir et al. [19] experimentally evaluated a reduction in tensile strength and delamination damage of flax fiber-reinforced composites during the drilling process. Khashaba and El-Keran [20] experimentally and analytically investigated the impact of machining parameters on the thrust force and delamination during drilling of a thin woven GFRP. Ekici et al. [21] studied the impacts of cutting parameters on the thrust force, surface roughness, and dimensional accuracy of Al/10B4C and Al/10B4C/5Gr composites. Geier et al. [22] developed a comprehensive review on advanced cutting tools and technologies for drilling CFRP composites. Cadorin et al. [23] found that the reinforcement of the composite material in the third direction removes the problem of delamination at the hole exit, even if the tool is worn and for the high feed rate used. Gemi et al. [24,25] studied the damage and surface quality of filament wound hybrid composite pipes with different stacking sequences during drilling. Khashaba et al. [26] experimentally explored the thrust force, torque, and delamination of GFRP composites during drilling processes with different machining parameters. Mudhukrishnan et al. [27] analyzed the thrust force and delamination in drilling GFR polypropylene composites using HSS twist, tipped carbide, and solid carbide drills. Bayraktar and Turgut [28] studied delamination at a hole entrance and exit during drilling of CFRP stacked on an aluminum plate under different cutting parameters. Ahmadi and Zeinedini [29] investigated the effect of drilling on the mode I delamination of GFRP laminates using experimental, theoretical, and numerical methods. Khanna et al. [30] studied the drilling performance of CFRP composites under dry and cryogenic environments. 

Considering thermal effects, Zitoune et al. [31] presented an original technique for the measurement of the machining temperature. This technique is based on in situ instrumentation with an optical fiber with Braggs sensors for monitoring, in real-time, the temperature generated when drilling thick 3D woven composites. Fu et al. [32] explored the drill exit temperature characteristics in drilling UD and MD CFRPs using a microscopy infrared imaging system. Erturk et al. [33] studied the effects of the cutting temperature and drilling parameters (drill bits, feed rate, and spindle speed) on the delamination of GFRP composites. Xu et al. [34] inspected the drilling forces/temperatures and the wear signatures of tools during drilling of multilayer CFRP/Ti6Al4V. Zhang et al. [35,36] predicted novel fiber fracture criteria in the machining process of CFRP by analyzing the effects of the axial force and hole exit temperature on the formation of hole exit surface damage.

At the microscopic level, Tang et al. [37] developed a 3D finite element model to examine the chip formation and delamination in the drilling of CFRP composites. Murthy et al. [38] used a system dynamic approach and Taguchi method to evaluate the influence of drilling parameters on the thrust force developed during drilling of GFRP. Feito et al. [39] predicted the damage induced during the drilling of composite materials using multi-objective optimization analysis of cutting parameters for special geometry drills. Liu et al. [40,41] presented a delamination model based on superposition of linear fracture mechanics capable of predicting a critical thrust force of aramid fiber-reinforced plastics by a brad drill. Wang and Jia [42] performed a full factorial experiment and utilized an artificial neural network for drilling CFRP with different drilling parameters to express the thrust force and delamination factor as a function of drilling parameters. Shahri et al. [9] exploited modified Mindlin–Reissner plate theory in conjunction with EFM in mixed mode loading conditions to predict the critical thrust force during the drilling process. Jai et al. [43] presented an analytical study of delamination damage and a delamination-free drilling method of CFRP composites. Bai et al. [44] and Wang et al. [45] proposed a novel mechanical model to predict a drilling thrust force with tool wear effects of unidirectional CFRP and a CFRP/Al stack. Khashaba et al. [46] studied the influence of the drill bit point angle on the generated heat on a woven GFRP composite. 

More than 60% of the drilling components used are rejected in the assembly stage due to delamination onset during the drilling process [47]. With increasing research works on this important topic, the number of rejected components has been progressively decreased, reflecting the importance of the present study. The most investigated parameters affecting the temperature induced in drilling FRP composites are the cutting speed and feed. The cutting temperature can be increased by increasing the cutting time (reducing feed) and increasing the thrust force (increasing feed). Therefore, in the present work, to avoid the opposite effects of the feed on the cutting temperature, the cutting time was increased at the same cutting conditions (feed and speed) via increasing the laminate thickness, which has not been investigated yet. Accordingly, three woven GFRE laminates were fabricated with the same fiber volume fractions (≈40%) and different thicknesses (2.6, 5.3 and 7.7 mm) by varying the number of woven glass layers (8, 16 and 24).

According to the knowledge of the authors and the literature review, investigation of the thermomechanical/failure behavior of woven GFRP composites laminated with different thicknesses under the drilling process with different feeds and speeds and including the heat-affected zone has not been addressed elsewhere. Therefore, the current article aims to fill this gap by investigating the temperature generated during the dry drilling of GFRP composites laminated by a cemented carbide drill bit with a 6 mm diameter and 118° point. The flank heat of the cutting tool was measured via instrumented drills, whereas the temperature of the heated zone around the hole was measured using a thermal imaging IR camera. The impact of machining parameters on the generated heat during drilling, and on the thrust force, torque, and delamination, was evaluated experimentally and validated numerically by multi-variable regression analysis.

## 2. Experimental Works

### 2.1. Specimen Preparation

Three woven GFRP composite laminates with varying thicknesses were manufactured using the hand lay-up technique. The polymer (epoxy) matrix was Araldite LY5138-2 and Hardener HY5138 (Sigma-Aldrich, Darmstadt, Germany). Symmetric lay-ups of orthogonal balanced woven fabric composites with thicknesses of 2.6, 5.3 and 7.7 mm were manufactured, respectively, from 8, 16 and 24 layers of E-woven roving glass fiber (3.5 yarns/cm for the warp and weft fibers). The cutting of glass fiber layers was through the warp and weft threads to ensure the right angles of all layers. The fiber volume fractions of the fabricated GFRP laminates were calculated by Equation (1) and are presented in Table 1.

(1) $$V_{f}=\frac{n\times A_{w}}{\rho_{f}\times t}$$

where *V_f_* is the fiber volume fraction, *n* is the number of layers, *A_w_* is the areal weight of the fabric, *t* is the thickness of the product, and $\rho_{f}$ is the fiber density.

### 2.2. Mechanical Characterization

According to ASTM D 3039, a series of standard ASTM tensile tests were performed to characterize the mechanical properties of the fabricated materials using the servohydraulic testing machine model Instron 8803 (500 kN) and 8872 (10 kN). The test specimens were cut to the standard dimension using a CNC abrasive waterjet machine to eliminate the heat generated by conventional machining processes. The specimens were loaded at a test rate of 1.0 mm/min to eliminate any source of dynamic effects. The longitudinal and transverse strains were measured using 4-channel data acquisition (DAQ) model 9237 NI. For each test, five samples were evaluated, and the average value is presented in Table 2.

### 2.3. Drilling Experimental Setup

**Figure 1:**
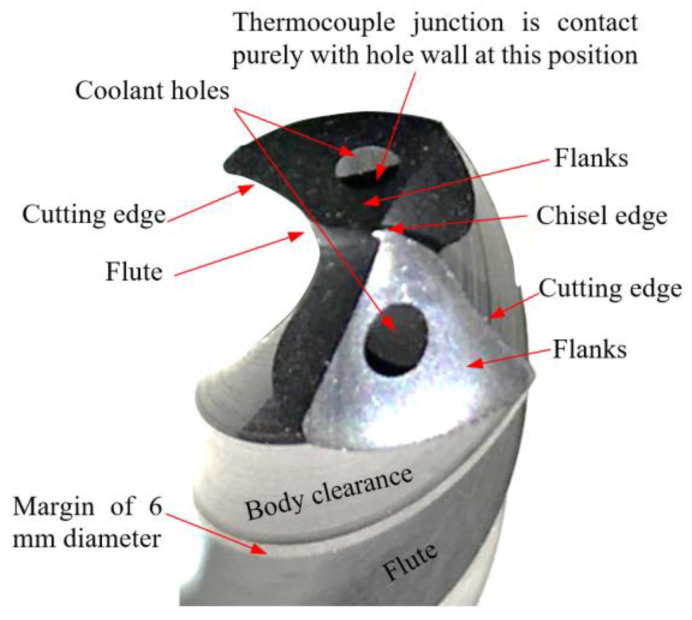
Different elements of the cemented carbide drill.

Drilling tests were conducted under dry cutting conditions using a CNC milling machine model “Deckel Maho DMG DMC 1035 V, ecoline”. Two flute-twist drills manufactured from special ultra-fine cemented carbide particles were used for efficient cutting, with excellent toughness and abrasion resistance. As provided by the manufacturer (Zhuzhou Best for Tools Co., Ltd., Zhuzhou, China), the details about the drill’s geometries are illustrated in Table 3. The drills were provided with two internal coolant holes of 0.6 mm diameter. Three identical drills were used in this study. The total cutting time for each drill did not exceed 4 min, which is too small to induce wear in the cemented carbide drill. The different elements of the drill, which are repeated in the Results and Discussion section, are illustrated in Figure 1.

**Figure 2:**
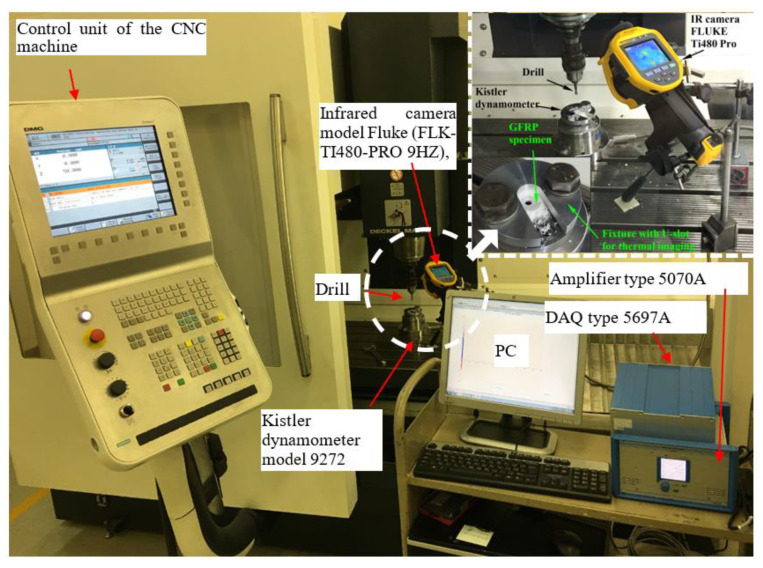
Drilling setup using CNC machine model Deckel Maho DMG DMC 1035 V.

The drilling tests were implemented on 36.6 mm × 36.6 mm specimens prepared from composite laminates using an abrasive water jet machine. The experimental setup with dynamometer–fixture–workpiece assembly is illustrated in Figure 2. Thrust force and torque data were recorded with a Kistler 9272. For the drilling parameters, a full experimental design was used through spindle speed (*N*), feed (*f*), and laminate thickness (*t*), as illustrated in Table 4. Three tests were performed for each machining factor. 

The temperature was measured using two different techniques. In the first technique, two K-thermocouple models TL0201 were embedded in coolant holes near the drill’s cutting edge. Each thermocouple contained two wires, each of 0.1 mm diameter. The wires were isolated, and thus the package diameter was 0.35 mm, which can fit in the drill’s cooling holes (0.6 mm) easily. Thermocouple junctions were in contact purely with the coolant hole walls by transition fit of an aluminum wire of 0.25 mm diameter and 15 mm length. Therefore, the temperature was transferred to the thermocouple junctions through pure contact with the hole wall and the higher thermal conductivity aluminum wire (210 W/m °C). The thermocouple can measure temperature up to 200 °C. Each analog thermocouple signal was converted to a digital signal using two USB data acquisition NI USB-TC01 modules connected to the PC. The temperature variation during the drilling process was monitored online and recorded using National Instruments LabVIEW Signal Express software. In this method, the instrumented drill was mounted by four independent jaw chucks, which were fixed on the dynamometer. The specimen was clamped firmly to the machine spindle using a special fixture.

In the second technique, the specimen was clamped firmly on the dynamometer using a special fixture, as shown in Figure 2. The fixture was designed with a U-slot of 20 mm width to measure the temperature induced in the heated zone using an infrared (IR) camera model FLUKE Ti480 Pro, which has a 640 × 480 resolution and a temperature measurement range from ≤−20 to +800 °C. The infrared camera was placed at 260 mm from the hole center and at an angle of 60°, as shown in Figure 2. For the two methods, the backplate under the test specimen had a 20 mm central hole diameter. The thermal imaging of the IR camera was calibrated using a mercury-in-glass thermometer immersed in boiling water, which resulted in excellent tolerance. The recorded video for each test was analyzed using SmartView v4.3 software. The software exported the temperature trend over time for the range of frames in the video. The data were imported into an Excel file to create a new data query. The data were treated via several steps to obtain the relationship between the number of frames/time (9 frames/s) and the measured temperature for the selected points.

### 2.4. Delamination Characterization

The peel-up and push-out surface delaminations were measured using the AutoCAD technique developed earlier [12,14,15]. This technique is suitable for quasi-transparent composite materials in which the drilled specimen was scanned using a high-resolution flatbed color scanner model Epson “V370, 4800 × 9600 dpi”. The transmitted light to the delaminated or damaged zone makes it brighter and easily distinguished from the undamaged area. The image was analyzed using CorelDraw software, by which the image was magnified to determine the delamination size within 10^−3^ mm. The delamination factor was defined as

(2) $$F_{d}=\frac{D_{max}}{D_{nom}}$$

**Figure 3:**
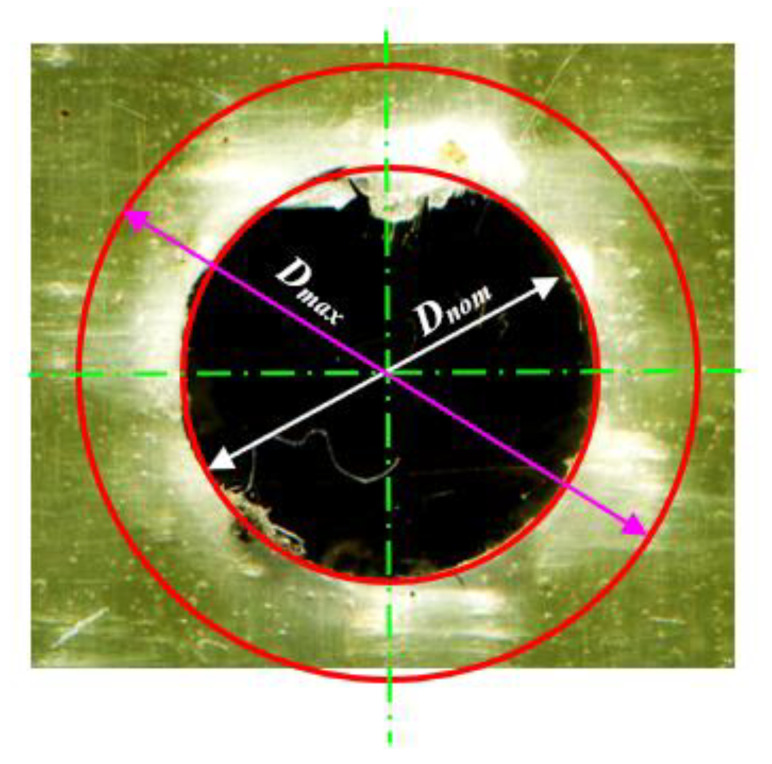
Push-out delamination in drilling GFRP composites at 400 rpm and 0.05 mm/r.

*F_d_* is the delamination factor, and *D_max_* is the maximum delaminated diameter drawn from the centerline of the hole nominal diameter (*D_nom_* = 6 mm), Figure 3.

## 3. Results and Discussion

### 3.1. Evolution of Thrust Force and Temperature

**Figure 4:**
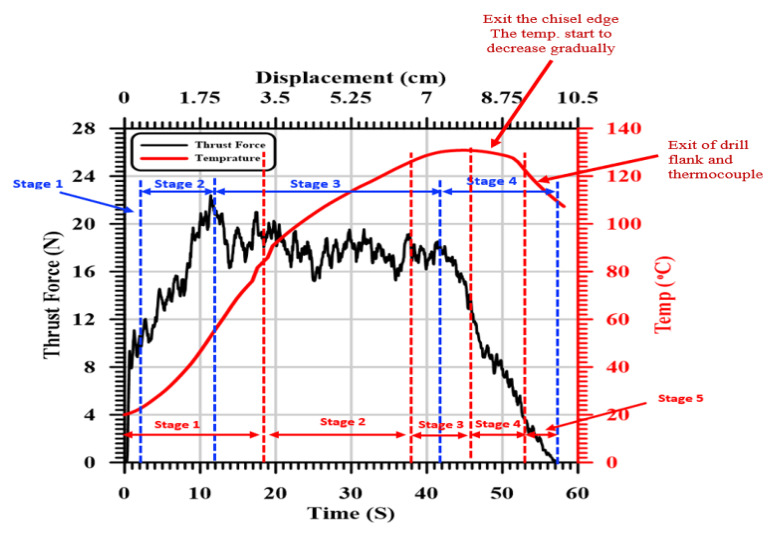
Evolution of thrust force and the induced temperature vs. time (displacement) in drilling GFRP with 7.7 mm thickness at 400 r/min and 0.025 mm/r.

Figure 4 illustrates the evolution of the thrust force and temperature concerning the cutting time and displacement during drilling of a woven GFRP composite with a thickness of 7.7 mm at a 400 r/min cutting speed and 0.025 mm/r feed. As it is shown, the thrust force and temperature vs. time can be categorized into four and five different stages, respectively, as follows: 

The GFRP composite behaved elastically in the first stage up to a thrust force of about 12 N (around 52% of the maximum *F_t_*) within 2 s (2 × 400 × 0.025/60 = 0.33 mm) at the entry of the chisel edge into the workpiece. At this stage, the chisel edge, with zero speed at its center, does not cut; instead, it extrudes the material. Chandrasekharan et al. [48] and Khashaba et al. [12] reported that the average chiseling edge thrust force is about 53% of the total thrust force.

At the end of the first stage, the drill penetrated the workpiece surface layer, and the second stage was observed. In this stage, the thrust force increased from 12 N to its maximum value of 23 N as the drill moved to 1.8 mm. This distance equals the approach allowance that accounts for the drill point angle of 118° = (D/2)/tan(118/2) = 1.8 mm. During this stage, the uncut chip area increased with the increase in the cutting depth until the total engagement of the drill point was achieved. After this distance, the drill was fully engaged with the workpiece, and the third stage was initiated. During the first and second stages, the flank temperature increased sharply because of the rapidly increasing tool–workpiece contact/cutting area. 

In the third stage, the temperature increased with the increasing hole depth because of the increasing friction between the drilling margin and the machined surface. The accumulated temperature in the third stage increased with a lower rate (slope) compared to that of the first and second stages. The increase in the accumulated drill temperature was assisted by the lower thermal conductivity of the GFRP composites. Khashaba et al. [14,15] showed that the thermal conductivity of GFRP composites is very low (0.59 W/m °C) compared to steel (=53 W/m °C), brass (=109 W/m °C), and aluminum (=210 W/m °C). The thermal conductivity of the glass fiber is (8.67 W/m °C) higher than that of the epoxy resin (0.14 W/m °C). Therefore, the heat accumulation during cutting mostly occurs in the resin matrix. In addition, the glass fiber has a much higher glass transition temperature (*T_g_* = 550 °C) compared to the epoxy matrix. The *T_g_* of used epoxy is (60.61 °C), determined in the present work by differential scanning calorimetry (DSC). The lower thermal conductivity and *T_g_* of the epoxy matrix played a vital role in its softening and burning, and thus the measured machineability parameters such as the thrust force, torque, and delamination factor will be seen later. 

In the third stage, the thrust force decreased gradually, which may be attributed to a reduction in the stiffness of the specimen caused by the removal of material layers under the drill and softening of the material due to the increasing cutting temperature. Through this stage, the temperature increased linearly until 6.7 mm after 38 s. At this point, there was an equilibrium balance between the energy generated by friction and the energy stored in the drill and workpiece. Therefore, the temperature remained constant as the drill moved from 6.7 to 8.3 mm (the third temperature). 

The fourth stage started when the chisel edge of the drill just exited the specimen, causing a higher reduction in the thrust force by about 50%. Then, in the fifth stage, a gradual reduction in the thrust force and temperature was observed up to the end of the drilling cycle due to the gradual exit of the drill cutting edges, as shown in Figure 4. 

### 3.2. Effect of Machining Variables on Temperature

**Figure 5:**
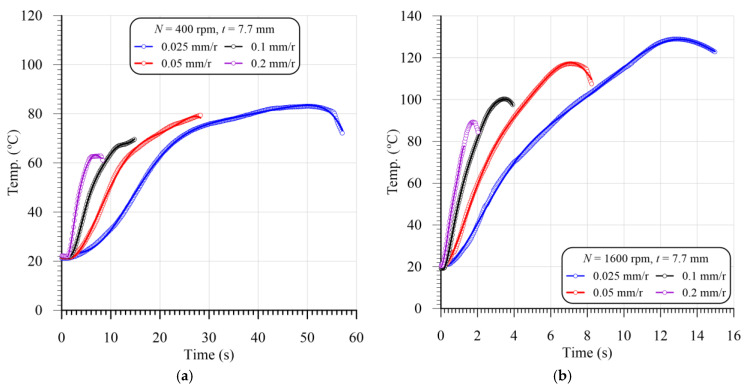
Representative samples of evolution of instrumented drill temperatures vs. cutting time in drilling GFRP composites with 7.7 mm thickness at different feeds and speeds of: (**a**) 400 rpm, and (**b**) 1600 rpm.

The temperature rising through the drilling of FRP composites can result in matrix burnout, debonding of the fiber/matrix interface, or even a glass transition of the heated zone, which severely deteriorates the composite materials’ quality and properties [34]. Figure 5a,b show a representative sample of the temperature evolution using the instrumented drills vs. cutting time in drilling GFRP with a 7.7 mm thickness at different feeds and speeds. It is obvious from Figure 5a,b that the induced temperature in drilling GFRP composites increased with the increasing cutting speed and feed. The temperature was sharply increased with time at the higher cutting speed of 1600 rpm, Figure 5b, relative to those drilled at 400 rpm, Figure 5a. This sharp increase is attributed to the friction increase between the abrasive glass fibers and the carbide drill with the increasing cutting speed. Additionally, severe debonding at the fiber/matrix interface can further increase the drilling temperature at higher cutting speeds due to the friction increase in the carbide drill with unsupported abrasive glass fibers, as reported by Yaşar and Günay [49]. 

**Figure 6:**
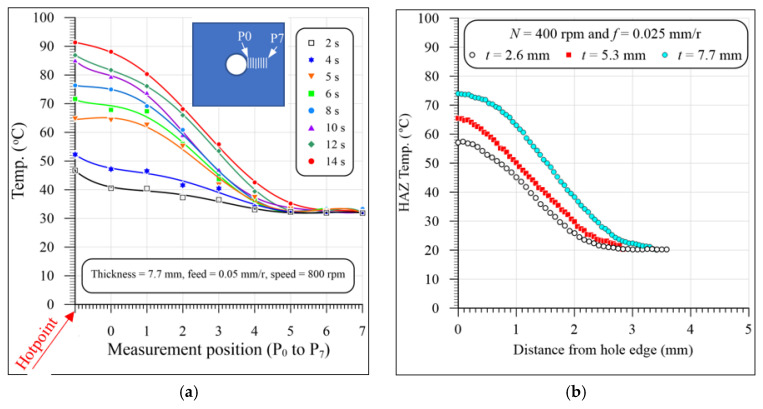
Representative sample of temperature distribution of the heated zone: (**a**) during cutting process (time) at 800 rpm, and 0.05 mm/r, and (**b**) effect of specimen thickness at 400 rpm, and 0.025 mm/r.

Figure 6a,b show the temperature distribution of the heated zone around the drilled hole. The distance from P_0_ to P_7_ is about 3.5 mm. The temperature of the heated zone increases rapidly during the cutting process (time). The hotpoint is the maximum recorded temperature by the IR camera during the drilling processes. This point can be the chip’s temperature or the drill bit temperature recorded during exiting the work. The results in Figure 6a show that the heated zone temperature continuously increases when advancing the drill toward exiting the work.

Figure 6b shows representative samples of the temperature distribution in the HAZ of the GFRP composites with different thicknesses at a speed of 400 rpm and feed of 0.025 mm/r. The results in Figure 6b show that the temperature of the HAZ was sharply decreased as it moved away from the hole edge because of the lower thermal conductivity of the GFRP composite laminates. The temperature reached a room temperature of about 20 °C after being about 2.8 mm, 3 mm, and 3.4 mm away from the hole edge of the composite laminates with thicknesses of 2.6 mm, 3.5 mm, and 7.7 mm, respectively. Merino-Pérez et al. [50] found that the temperature decreased from 360 to 50 °C after being 3.5 mm from the hole edge in drilling FRP composites at speeds ranging from 50 to 200 m/min. They measured the temperature distribution using thermal imaging and thermocouples impeded at different distances around the hole. 

**Figure 7:**
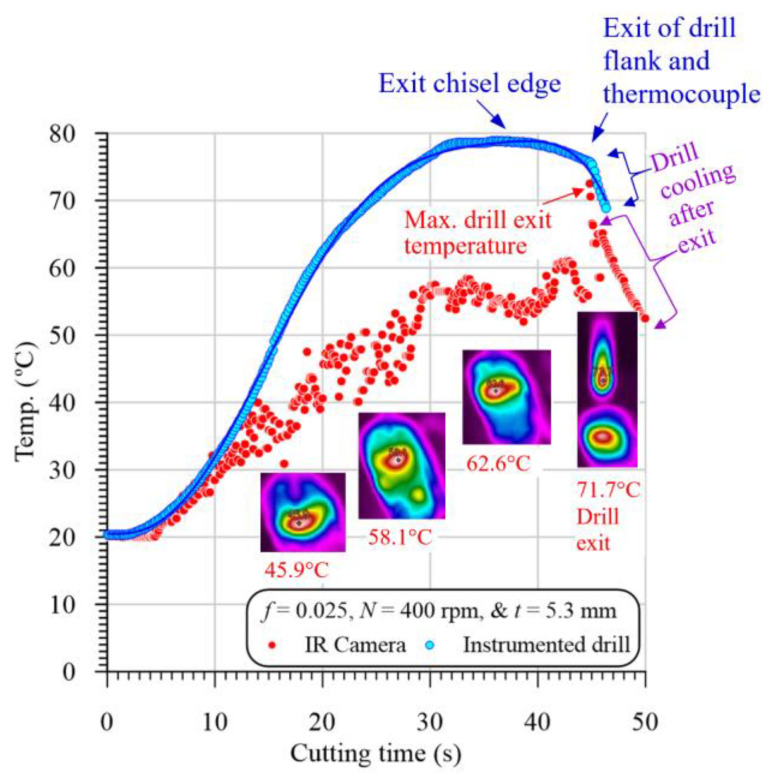
Representative sample of evolution of temperature at hole edge (P0) vs. cutting time in drilling GFRP with 5.3 mm thickness at 400 rpm and 0.025 mm/r.

Figure 7 illustrates a representative sample of the temperature vs. cutting time evolution in drilling GFRP with a 5.3 mm thickness at 400 r/min and 0.025 mm/r. The temperature was measured by both the instrumented drill and the IR camera. It is clear from Figure 7 that the measured temperature values by the two methods at the first 10 s are almost identical. This identical measurement is attributed to the drill entry, and the chisel edge with zero speed at its center does not actually cut, but, instead, it extrudes the material. Therefore, the camera records the drilling temperature that equals that measured using the instrumented drill. A similar observation was reported by [34] in drilling CFRP/Ti6Al4V stacks. 

After 10 s, the drill point is cut only 1.67 mm (10 × *f* × *N*/60) from its approach allowance (1.8 mm). Therefore, in the first 10 s, the camera measures the drill point temperature, which approximately equals that measured by the instrumented drill. As the drill penetrates the specimen, the drill–work interaction zone becomes unaccessible, and thus the IR camera measures the temperature of the HFZ, which is lower than that of the drill point measured by the instrumented drill, as shown in Figure 7. At the drill exit of the workpiece, the IR camera records a sudden increase in the temperature. This increase is because the camera always records the highest temperature in the drilling zone. This result indicates that the drill point temperature (72 °C) is higher than that of the hole edge (62 °C) by about 10 °C, as shown in Figure 7. It is also evident that the maximum temperature recorded by the IR camera is lower than that of the instrumented drill. This difference is attributed to the IR camera not directly measuring the tool–work interaction zone during the drilling process. Accordingly, the drill point was partially cooled during the exit of the machined hole. Xu et al. [34] calibrated the IR camera’s temperature by adding a compensation value equal to the difference between the measurements by the two methods. However, this method is not accurate for the following reasons:

- The difference is increased with the specimen thickness, where the drill takes a longer time during the exit out of the specimen and thus loses more heat than the thinner one.
- For the same specimen thickness, the difference between the measured temperatures by the two methods is decreased with the increasing feed values because of the decreasing cutting time and, thus, decreasing measuring time between the two methods.
- In some cases, the hot chips were dropped out of the drill flutes and dispersed on the specimen surface, and thus the measured temperature cannot be calibrated.

Therefore, in the current analysis, the temperature of the instrumented drill was used to construct the different relationships with the cutting variables.

**Figure 8:**
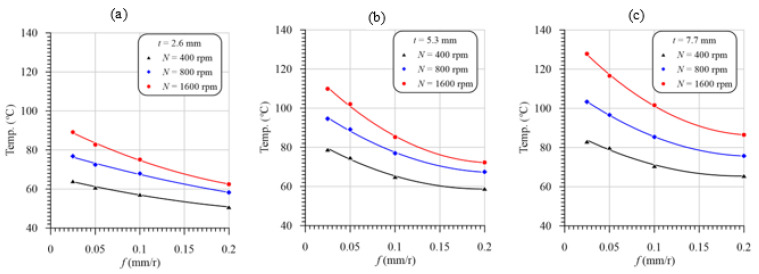
Temperature vs. feed at different speeds and laminate thicknesses of (**a**) 2.6 mm, (**b**) 5.3 mm, and (**c**) 7.7 mm.

**Figure 9:**
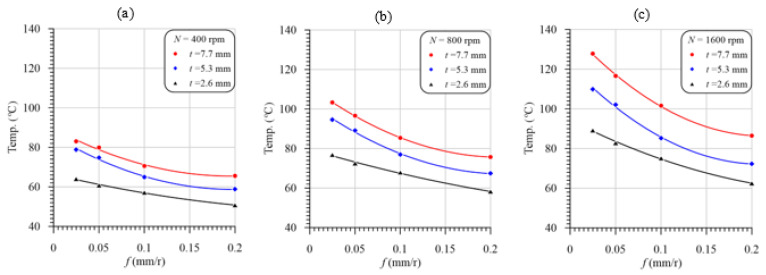
Temperature vs. feed with different laminate thicknesses and speeds of (**a**) 400 rpm, (**b**) 800 rpm, and (**c**) 1600 rpm.

Figure 8a–c shows the variation in the drill temperature vs. feed with varying cutting speeds in drilling GFRP composites with thicknesses of 2.6, 5.3 and 7.7 mm, respectively. The data in Figure 8 were redrawn to illustrate the effect of the laminate thickness on the temperature rise in drilling GFRP composites, as shown in Figure 9. The second-order polynomial equation fits very well the measured temperature curves of the GFRP laminates. It is evident from these figures that for the investigated cutting speeds and laminate thicknesses, the peak flank temperature is inversely proportional to the feed because of the decreasing cutting time. In contrast, at the same feed values, the maximum temperature curves were observed at the maximum speed and laminate thickness, as shown in Figure 8 and Figure 9, respectively. As it is shown, at a feed of 0.025 mm/r and thickness of 2.6 mm, the temperature increased from 60 to 95 °C by increasing the speed from 400 to 1600 rpm, which means the temperature increase is 55%. Increasing the thickness from 2.6 to 7.7 mm has a significant effect on the drill point temperature. The thickness increase from 2.6 to 7.7 mm increases the temperature from 95 to 127.5 °C, at 1600 rpm and 0.025 mm/r.

The drilling temperature increase with the cutting speed is attributed to the frictional heat increase at the tool rake face and the drill flanks [51]. The friction between the machined surface and the drill margins and between chips and flutes is another reason for the increasing measured temperature with the increase in the drilling speed, whereas the increasing measured temperature with the laminate thickness, Figure 9, was due to the increasing cutting time and, thus, the accumulated friction heat at the drill point. The increased cutting temperature in drilling a GFRP laminate with a thickness of 5.3 mm is in the range of 13–23% compared to that of the 2.6 mm-thick GFRP laminate, as shown in Figure 8a,b. The temperature increase in the thicker laminate (7.7 mm) was approximately doubled (23–43%), as shown in Figure 8c. It is evident from Figure 8c that the maximum drilling temperature is (128 °C) obtained at the maximum speed, lower feed, and maximum laminate thickness. Therefore, the drill speed and the laminate thickness are the most significant parameters on the temperature rather than the feed. This conclusion is consistent with the observation noticed [36]. 

### 3.3. Effect of Machining Variables on Thrust Force

**Figure 10:**
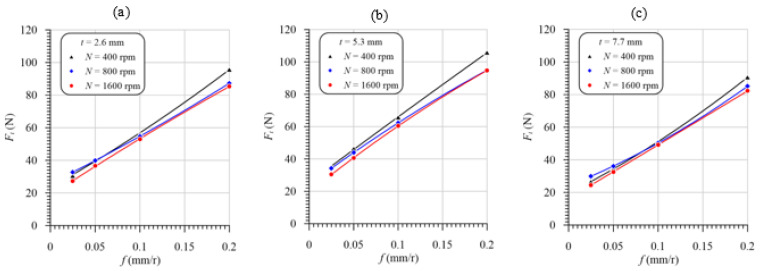
Thrust force vs. feed at different speeds and laminate thicknesses of (**a**) 2.6 mm, (**b**) 5.3 mm, and (**c**) 7.7 mm.

**Figure 11:**
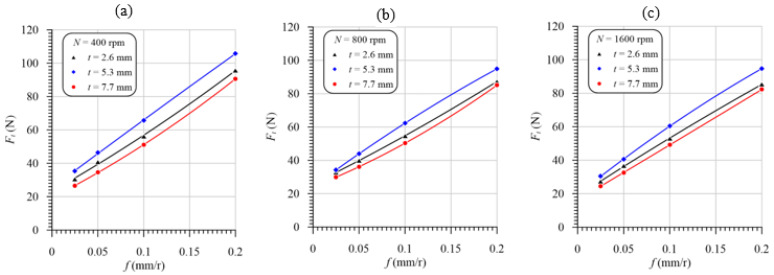
Thrust force vs. feed at different laminate thicknesses and speeds of (**a**) 400 rpm, (**b**) 800 rpm, and (**c**) 1600 rpm.

The effect of the feed and speed on the maximum thrust force and torque in drilling GFRP laminates with thicknesses of 2.6, 5.3 and 7.7 mm is presented in Figure 10. The measured thrust force decreases slightly at different laminate thicknesses as the speed increases. Comparing Figure 8a–c with Figure 10a–c, respectively, the increase in the temperature accompanied by the increasing drill speed leads to a decreasing thrust force in the same order of thicknesses. This behavior can be demonstrated by comparing Figure 9a–c with Figure 11a–c. It is obvious from Figure 9 that the thicker specimens (7.7 mm) exhibit the highest temperature and lowest thrust force relative to those of specimens with a thickness of 5.3 mm, as shown in Figure 11. Xu et al. [34] attributed the reduction in the thrust force with the increasing drilling speed to the thermal softening of the FRP composites. Although the thinner specimens (2.6 mm) have the lowest temperature, their thrust forces are lower than those of the 5.3 mm-thick specimens. This behavior is attributed to the lower stiffness of the thinner specimen, which is more effective than reducing the stiffness of specimens with a 5.3 mm thickness because of the higher temperature and softening. 

With smaller thicknesses at 2.6 mm, by increasing the cutting speed from 400 to 1600 rpm, the maximum thrust force is decreased by 15%. However, with the other thicknesses, this reduction is less than 15%. Through all cases shown in Figure 10, it is obvious that the higher the feed, the higher the thrust force. Therefore, there is a significant proportional effect of the feed on the thrust force compared to the cutting speed. This result is attributed to increasing the cross-sectional area of the uncut chip (*A* = *D* × *f*/4) with increasing feed. 

### 3.4. Effect of Machining Variables on the Torque

**Figure 12:**
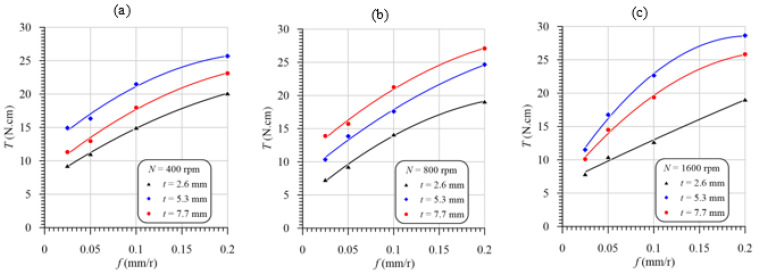
Torque vs. feed at different laminate thicknesses and speeds of (**a**) 400 rpm, (**b**) 800 rpm, and (**c**) 1600 rpm.

It is evident from Figure 12 that the cutting temperature was increased with the increasing feed at different speeds and laminate thicknesses. This temperature increase is attributed to the friction force increase between the machined surface and both drill flanks and margins and between the chip and flutes. Increasing the cross-sectional area of the uncut chip (*A* = *D* × *f*/4) is another reason for the increasing friction force on the rake face and the tool point’s flank face, thus increasing the torque. The engaged drill body length just before the exit of the chisel edge of the drill point of the laminate with a 2.6 mm thickness is 0.8 mm (=2.6 mm—approach allowance, 1.8 mm), very small compared to the lengths of 3.5 mm and 5.9 mm of the laminate thicknesses of 5.3 mm and 7.7 mm, respectively. Hence, at the same cutting speeds and feeds, the lowest torque is found for the composite laminate with the lowest thickness (2.6 mm) because of the decreasing friction area between the drill margins and the machined hole wall surface. Additionally, the GFRP laminate with a 2.6 mm thickness has a lower induced temperature, Figure 9, and thus lower thermal expansion. Increasing the drill thermal expansion can significantly increase the friction between drill margins and the machined hole wall surface. In addition, the friction between the chip and the drill flute is decreased with the decreasing specimen thickness. For this reason, the torque of the GFRP laminate with a 5.3 mm thickness is higher than that of the 2.6 mm-thick specimens, as shown in Figure 12a–c. 

The largest induced temperature was observed in drilling specimens with a 7.7 mm thickness, as shown in Figure 9. This high temperature results in a higher thermal expansion of the drill and, thus, increases margins’ friction torque, as shown in Figure 11b. The higher temperature in drilling specimens with a 7.7 mm thickness can lead to softening the epoxy matrix. The softer materials can work as a lubricant and thus reduce the torque, as shown in Figure 12a,c. Therefore, it can be concluded that the different positions of the drilling torque of the specimens with the largest thickness (7.7 mm) relative to those of 5.3 mm thickness are attributed to it having the largest temperature, which can play opposite roles in the friction torque of the drill margins.

### 3.5. Effect of Machining Variables on the Delamination Factor

**Figure 13:**
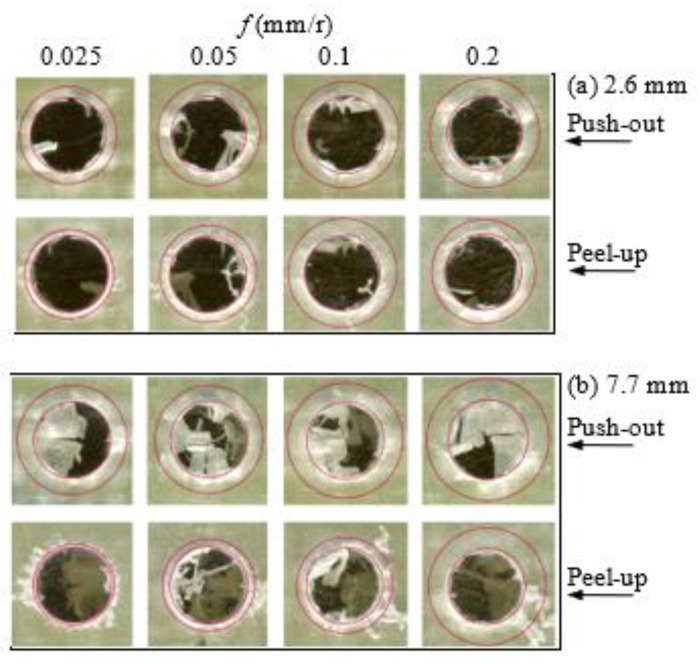
Representative samples of delaminations in the drilling of GFRP laminate at a speed of 1600 rpm: (**a**) *t* = 2.6 mm and (**b**) *t* = 7.7 mm.

Delamination affects the structural integrity and reliability of FRP composites, and thus the economic impact of the delamination induced in drilling is significant, particularly considering the various stages associated with the component when it reaches the final assembly line [20]. Delamination induced in drilling FRP composite laminates exhibited a complex failure mode consisting of a combination of mechanical and thermal damage. The delamination may occur at the entry (peel-up) and exit planes (push-out) of the composite laminate. Peel-up delamination occurs due to two fracture modes: mode I, in which opening cracks occur because the fibers of the top plies are not cut sufficiently due to the unfavorable cutting conditions; mode II, where, when the cutting edges of the twist drill encounter the composite laminates, a peeling force is generated through the slope of the drill flute and separates the top plies, causing delamination [52]. Push-out delamination of the bottom surface occurs through both modes, mode I and mode II, since the drilled composite material is subjected to an axial force and bending. Figure 13 shows some representative samples of peel-up and push-out delaminations at different feeds, speeds, and laminate thicknesses. Push-out delamination is higher and more critical than peel-up because of the lack of backup support, compensating for the thrust force during drill penetration [14,15,46]. On comparing Figure 13a with Figure 13b, it is evident that at the same cutting condition, the push-out delaminations of the GFRP laminate with a 7.7 mm thickness are higher than those of specimens with a 2.6 mm thickness and accompanied by edge chipping, spalling, and uncut fibers. There are excessive uncut fibers spread outward because the fibers bend or move away from the path of the advancing tool. This behavior is attributed to the highest temperature induced in the drilling of the thicker laminate (7.7 mm), as shown in Figure 9, that leads to softening the matrix and hence bending the last layer instead of cutting by the drill edges. Chipping and spalling defects are also observed at the hole’s entry, thus decreasing the surface quality, as shown in Figure 13b. 

**Figure 14:**
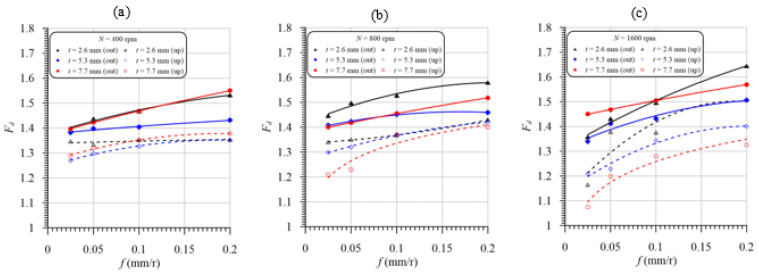
Delamination factor vs. feed of different laminate thicknesses at speeds of (**a**) 400 rpm, (**b**) 800 rpm, and (**c**) 1600 rpm.

Effects of feed and laminate thickness on peel-up and push-out delamination factors in drilling GFRP composites at speeds of 400, 800 and 1600 rpm are presented in Figure 14a–c, respectively. It is evident from Figure 14 that peel-up and push-out delamination factors are increased significantly by the increasing feed as a result of the increasing thrust force, Figure 11. For all cases, the peel-up delamination factor is less than the push-out delamination, consistent with that previously reported by [20,53]. From Figure 14c, the push-out and peel-up delaminations are sharply increased with the feed due to drilling at the highest speed and temperature, Figure 9. Although the thrust force of the specimen thickness of 5.3 mm is higher than that of the specimens with a 2.5 mm thickness, the push-out and peel-up delaminations of the latter are higher than those of the former, as shown in Figure 11. This result is attributed to the lower stiffness of the thinner laminate and, thus, the higher bending deflection of the last layer compared to those of specimens with a 5.3 mm thickness. The delamination of the specimen with a thickness of 7.7 mm is higher than that of specimens with a 5.3 mm thickness. This result is attributed to the combination of mechanical and thermal damage in drilling a specimen with a 7.7 mm thickness, which has the highest cutting temperature, as shown in Figure 9. At the severe cutting conditions, with a higher speed (1600 rpm) and feed of (0.2 mm/r), the delamination of the GFRP laminate with a thickness of 2.6 mm is higher than that of specimens with a 7.7 mm thickness. This result is attributed to the higher thrust force and lower stiffness of the thinner laminate. It can be concluded that the feed and laminate thickness have the largest contribution to the delamination damage due to the increasing thrust forces and temperature, as shown in Figure 9. A similar observation was reported by Mohan et al. [54]. Their study predicted that peel-up delamination is influenced by the specimen thickness and cutting speed. At the same time, push-out delamination is influenced by the specimen thickness and feed. 

At the beginning of the drilling operation, the thickness of the laminated composite materials can withstand the cutting force. As the tool approaches the exit plane, the stiffness provided by the remaining plies may not be enough to bear the cutting force, causing the lamina to separate, resulting in delamination. The delaminations that occur during drilling severely influence the mechanical characteristics of the material around the hole. It is necessary to determine the optimum conditions (feed, cutting speed, and material thickness) for a particular machining operation to avoid delaminations. Therefore, the optimization technique and multi-variable regression were conducted, and the findings are presented in the next section to predict the optimum drilling conditions. 

### 3.6. Effects of Cutting Time on Temperature, Thrust Force, Torque, and Delamination

**Figure 15:**
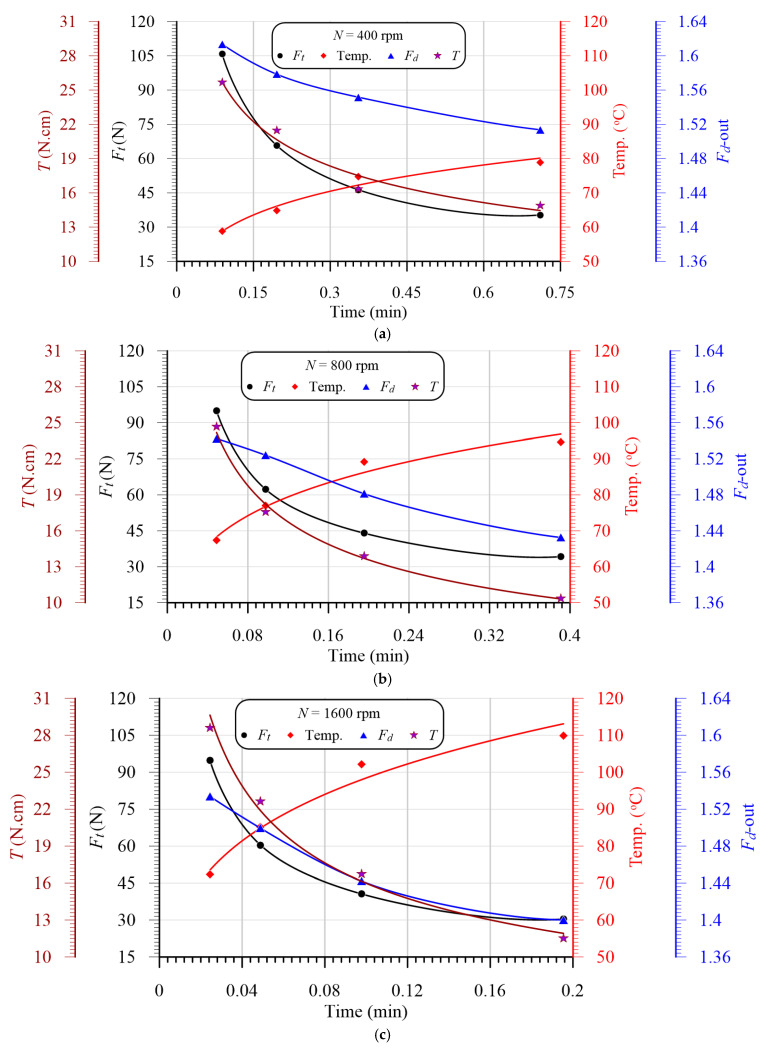
Variation in thrust force, delamination, and temperature vs. the cutting time at different speeds. (**a**) N = 400 rpm; (**b**) N = 800 rpm; (**c**) N = 1600 rpm.

Figure 15a,c are exploited to present the coupling effects between the mechanical thrust force, delamination parameter, temperature, and cutting time at speeds of 400 rpm, 800 rpm and 1600 rpm, respectively. It is shown that the thrust force and delamination have the same behaviors, rather than the temperature, as the variation in the drilling time, which assures that the delamination is proportionally dependent on the thrust force and inversely dependent on the temperature, which may lead to softening. Therefore, the thrust force and temperature have a coupling effect on the delamination ratio, which will be investigated statistically in the next section. From Figure 15, it can be concluded that, by increasing the drilling time, the temperature of the drill and chip increased, and the thrust forced decreased, in exponential forms.

## 4. Statistical Analysis

In recent years, considerable attention has been paid to using multiple regression models for correlating machinability parameters with machining conditions in drilling fiber-reinforced composites [14,15,55,56]. Design of experiment (DoE) methods have been used extensively in investigating the significance of cutting condition factors on the delamination of fiber composites during the drilling process. Abhishek et al. [57] presented a regression model using a harmony search (HS) algorithm to evaluate performance characteristics in the drilling of CFRP by using a TiAlN-coated solid carbide drill bit. Box–Behnken design with a simulated annealing algorithm was used to develop a regression model to control the thrust force and delamination in the drilling of graphene oxide/CFRP nanocomposites [58]. Di Benedetto et al. [59] employed an artificial neural network (ANN) and DoE for developing a prediction model of the energy absorption capability of thermoplastic composites. Much research has combined DoE and ANNs to develop prediction models. Damage to composite structures could be predicted using vibration data and a dynamic transmissibility ensemble with an auto-associative neural network [60,61,62,63]. Damage in a girder bridge was predicted using transmissibility functions as input data to artificial neural networks by [64]. 

As outputs of the drilling operation (responses), thrust force, torque, and temperature were measured during the experiment. In the present study, a factorial design was used to identify the main effects of three factors named feed, spindle speed, and workpiece thickness on the machinability responses mentioned above. The machining properties were measured according to the design of experiments for actual independent drilling process variables, with their levels illustrated in Table 4. 

### 4.1. Statistical Results

The primary objective for employing ANOVA was to investigate the significance of machining parameters affecting the machinability properties, including the thrust force, torque, cutting temperature, and delamination factor. The ANOVA results are summarized in Table 5. The effect of each parameter on the measured properties was evaluated by its contribution percentage to the total variation. The significant effect of the machining parameters on the machinability of the GFRP composite can be measured by the *p*-value. For most experimental work, a *p*-value less than 0.05 indicates the significance of the related factor for the response. Accordingly, all machining parameters have a significant effect on the measured temperature, as shown in Table 5. The largest contribution is from the speed (34.39%), followed by the feed (30.94%) and thickness (28.76%).

The contribution of the feed to the measured thrust force is about 95.38%, which is higher than that of the laminate thickness (3.04%). However, the effect of the laminate thickness is higher than the cutting speed (0.78%), which agrees with Figure 10 and Figure 11. The lower contribution of the speed is attributed to the indirect effect of the increasing temperature accompanied by the decreasing GFRP specimen stiffness on the measured force. The ANOVA results presented in Table 5 show that the torque is primarily affected by the feed (73.81%) and then the thickness (19.45%), while the effect of the speed is not significant (*p* = 0.778).

The feed is the most significant drilling parameter affecting the delamination factor (58.50%) due to its high effect on the thrust force (95.38%). The thickness of the laminate affects delamination by 17.86%. At the same time, the spindle speed has no significant effect on delamination, with a *p*-value of (0.1).

Since the drilling parameters were considered at multiple levels, in Table 4, quadratic mathematical models based on response surface methodology are developed to predict machinability properties, as shown in Table 6. The regression models were used to generate the response surface plots for all machinability properties. The results shown in Table 6 indicate that the predicted machinability properties have good agreement with the experimental results, as shown by the higher values of the coefficient of determination (R^2^).

**Figure 16:**
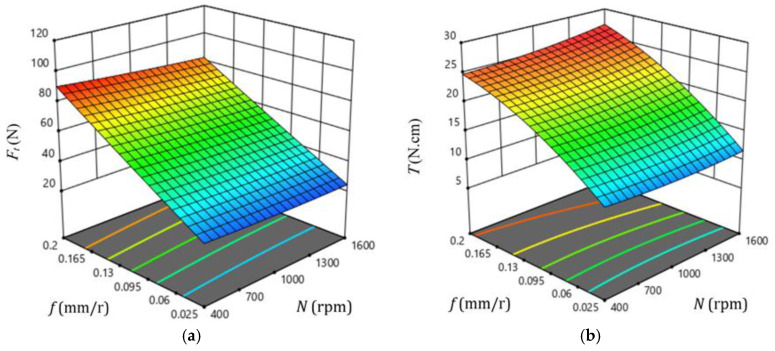

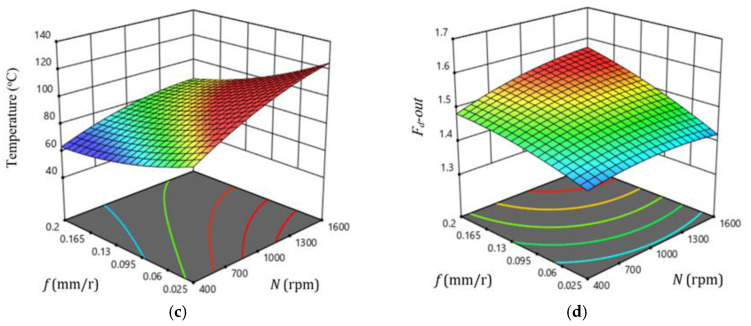
Response surface plots showing the effect of drilling parameters on the machinability properties of GFRP composite with a thickness of 7.7 mm: (**a**) *F_t_*, (**b**) *T*, (**c**) temperature, and (**d**) *F_d_*-out.

**Figure 17:**
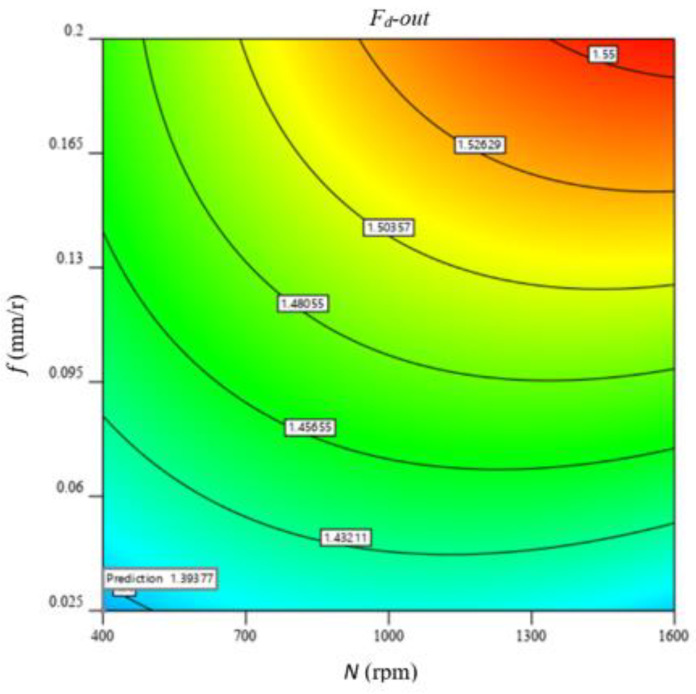
Contour plot of push-out delamination factor optimization at workpiece thickness of 7.7 mm.

Figure 16 illustrates 3D surface and contour plots of machinability responses vs. different drilling parameters of the GFRP composite with a thickness of 7.7 mm, as a representative sample. These plots can easily indicate the critical conditions for the predicted machinability properties. For example, at a feed of 0.2 mm/r, the critical thrust force and torque were observed at speeds of 400 and 1600 rpm, respectively, as shown in Figure 16a,b. Similarly, the critical temperature was observed at a feed of 0.025 mm/r and a speed of 1600 rpm, as shown in Figure 16c. Response surface analysis through Figure 16d indicates the minimum push-out delamination factor is observed at lower feed and speed values, as shown in the contour plot of Figure 17. The push-out delamination at any cutting condition can be predicted using the contour plot of Figure 17. 

### 4.2. Optimizing Delamination Factor

**Figure 18:**
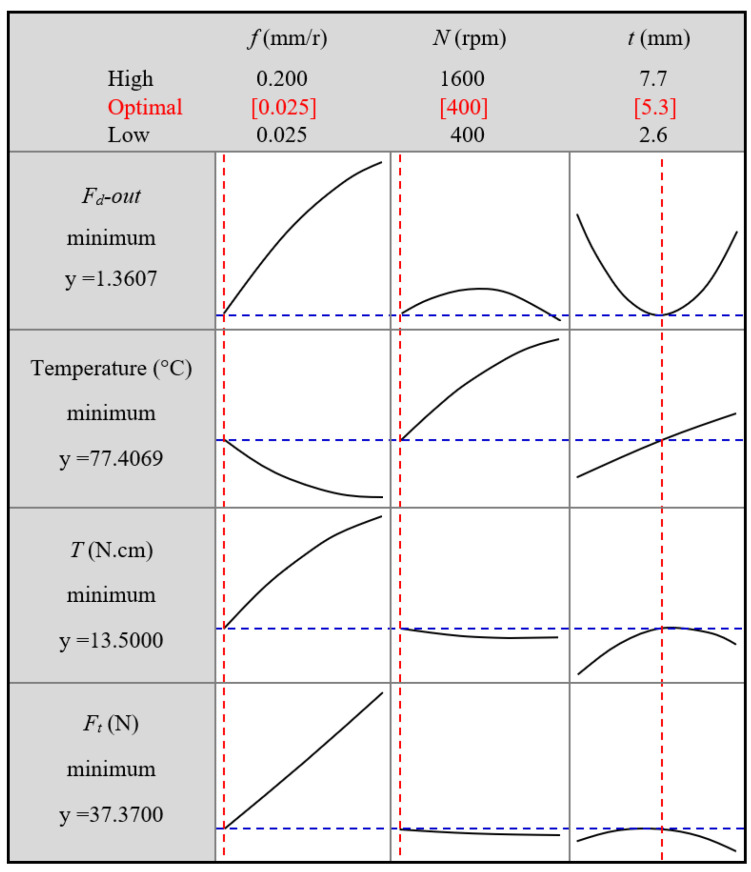
Optimum response according to different machining parameters.

The optimization function aims to minimize all machinability properties of drilling GFRP composites. The optimization plot in Figure 18 reveals that the optimal parameters are a feed of 0.025 mm/r, speed of 400 rpm, and material thickness of 5.3 mm, while it is observed that the optimal parameters for the minimum push-exit delamination, without respect to other machinability properties, are a feed of 0.025 mm/r, speed of 1600 rpm, and laminate thickness of 5.3 mm. This combination may produce the minimum push-exit delamination but is associated with the maximum temperature, as shown in the plot dedicated to temperature in Figure 18. 

## 5. Conclusions

The effect of machining parameters on the thermomechanical response of a woven GERP composite laminated under the drilling procedure was studied compressively in this article. The impact of machining parameters on the generated heat, thrust force, torque, and delamination in drilling GFRP composite laminates with different thicknesses was evaluated. The distributions of the surface temperature of the heat-affected zone (HAZ) and drill point temperature were investigated using a thermal infrared camera and instrumented drills with thermocouples. The main outcomes from this study can be summarized as follows:

➢ The IR camera is useful for characterizing the surface temperature of the HAZ, whereas the instrumented drill is more accurate for measuring the drill point temperature.
➢ The temperature of the HAZ was sharply decreased as it moved away from the hole edge due to the lower thermal conductivity of the GFRP composite laminates.
➢ The increase in the temperature occurs because increasing the drill speed leads to decreasing the thrust force.
➢ The thrust force and delamination have the same behaviors, rather than the temperature, as the variation in the drilling time, ensuring that the delamination is proportionally dependent on the thrust force and inversely dependent on the temperature.
➢ The thrust force and temperature have a coupling effect on the delamination ratio. By increasing the cutting time, the temperature increased, and the thrust force decreased, in exponential forms.
➢ At the same cutting condition, the push-out delaminations of the GFRP laminate with a 7.7 mm thickness were evidently higher than those of specimens with a 2.6 mm thickness and accompanied by edge chipping, spalling, and uncut fibers. This behavior was attributed to the highest temperature induced in the drilling of the thicker laminate, which leads to softening the matrix and hence bending the last layer instead of cutting by the drill edges.
➢ From the ANOVA results, all drilling conditions significantly influenced the generated temperature, while the feed and material thickness were found to make the largest contributions to the delamination effect. The optimal cutting conditions are a feed of 0.025 mm/r and a speed of 400 rpm when the drilling process is carried out on a GFRP laminate with a 5.3 mm thickness.
➢ The presented model can be used to predict the thrust force, delamination, and the generated temperature during the drilling procedure of GFRP, thus determining the optimum drilling conditions to generate a high-quality hole.

In the future, the present model will consider the thermomechanical behavior of composite structures with different reinforcement materials and different drill bit geometries and types.

## Figures and Tables

**Table 1:** The estimated fiber volume fraction.

*n* (Layers)	*A_w_* (g/m^2^)	*ρ_f_* (g/cm^3^)	*t* (mm)	*V_f_* (%)
8	324	2.5	2.59	40.0
16	324	2.5	5.25	39.5
24	324	2.5	7.73	40.2

**Table 2:** The mechanical properties of woven GFRP composites.

Poisson’s Ratio υ_12_ = υ_21_	Standard Deviation	Young’s Modulus (GPa) E11 = E22	Standard Deviation	Tensile Strength (MPa)	Standard Deviation
0.295	0.015	16.05	0.116	203.86	4.215

**Table 3:** Geometries of the cemented carbide drills.

D (mm)	Flute Length (mm)	Overall Length (mm)	Helix Angle	Rake Angle	Clearance Angle	Point Angle	Chisel Edge Length (mm)
6	28	66	30°	30°	12°	118°	0.3

**Table 4:** Levels of the variables used in the experiment.

Factors	Unit	Levels			
		1	2	3	4
Spindle speed, *N*	r/min	400 (7.5 m/min)	800 (15 m/min)	1600 (30 m/min)	
Feed, *f*	mm/r	0.025	0.05	0.1	0.2
Thickness of sample, *t*	mm	2.6	5.3	7.7	

**Table 5:** ANOVA results with the contribution of control factors’ effect on machinability responses.

Source	DF	*F_t_*	*p*-Value	*T* (N·cm)	*p*-Value	*F_d_*-Out	*p*-Value	Temp	*p*-Value
*f* (mm/r)	3	95.38%	0.000	73.81%	0.000	58.50%	0.000	30.94%	0.000
*s* (N·cm)	2	0.78%	0.040	0.12%	0.778	3.58%	0.100	34.39%	0.000
*t* (mm)	2	3.04%	0.000	19.45%	0.000	17.86%	0.000	28.76%	0.000
Error	28	0.79%		6.61%		20.05%		5.91%	
Total	35	100.00%		100.00%		100.00%		100%	

**Table 6:** Nonlinear regression model for machinability responses.

Response	Regression Equation
Thrust Force (N) R^2^ = 0.993	$\begin{array}{cc} F_{t}=2.08734 & -0.006772s-354.7068f+12.16301t\\ & -0.023699sf+0.000211st\\ & +0.53496ft+1.7899e^{-06}s^{2}\\ & +58.28871f^{2}-1.27964t^{2} \end{array}$
Torque (N·cm) R^2^ = 0.945	$\begin{array}{cc} T=-3.54528 & -0.005181s+100.9026f+5.54359t\\ & +0.012219sf+0.000278st\\ & +2.59726ft+1.30e^{-06}s^{2}\\ & -229.53008f^{2}-0.495062t^{2} \end{array}$
Drill Temperature (°C) R^2^ = 0.990	$\begin{array}{c} \mathrm{Temp.}=40.73943+0.045223s-161.85447f+\\ 4.93629t-0.081687sf+0.002271st-\\ 10.53577ft-1.4e^{-05}\;s^{2}+649.98743f^{2}-0.152154 \end{array}$
Delamination Exit R^2^ = 0.852	$\begin{array}{cc} F_{d}-out=1.55484 & +0.00006s+1.07382f\\ & -0.091623t+0.000381sf\\ & +4.7e^{-06}st-0.053205ft\\ & -4.89e^{-08}s^{2}-1.81211f^{2}\\ & +0.008641t^{2} \end{array}$

## Data Availability

All data available on request.
